# A rapid and nondestructive protocol for whole-mount bone staining of small fish and *Xenopus*

**DOI:** 10.1038/s41598-018-25836-4

**Published:** 2018-05-10

**Authors:** Hiromi Sakata-Haga, Maimi Uchishiba, Hiroki Shimada, Tsuyoshi Tsukada, Mayumi Mitani, Tomohiro Arikawa, Hiroki Shoji, Toshihisa Hatta

**Affiliations:** 1 Department of Anatomy, Kanazawa Medical University, Ishikawa, Japan; 2 Department of Obstetrics and Gynecology, Wakayama Medical University, Wakayama, Japan; 3 Department of Biology, Kanazawa Medical University, Ishikawa, Japan

## Abstract

Here we propose a new protocol for whole-mount bone staining, which allows the rapid preparation of highly cleared and nondestructive specimens. It only takes 3 days to complete whole procedure for small vertebrates, such as medaka, zebrafish, and *Xenopus* frogs. In this procedure, we used a newly developed fixative containing formalin, Triton X-100, and potassium hydroxide, which allows the fixation, decolorization, and transparentization of specimens at the same time. A bone staining solution containing alizarin red S with ethylene glycol and a clearing solution containing Tween 20 and potassium hydroxide also contributed the specificity and swiftness of this new system. As expected, although details of the skeletal system could be observed in specimens with high transparency, it was noteworthy that high-resolution fluorescence images acquired using zoom microscopes clearly delineated the shape of each bone. This new procedure would be expected to be widely used as a standard procedure for bone staining in the testing the developmental toxicity of chemicals and in the screening test of knockout or mutant animals.

## Introduction

Whole-mount bone staining has commonly been used to examine the morphology of skeletal system, especially for reproductive toxicology testing. Since the original procedure for whole-mount bone staining was first reported^1^, it has undergone various modifications. Staining bones with alizarin red S after clearing away the soft tissue with a solution containing potassium hydroxide (KOH) was widely used for many years as a standard method for bone staining in small vertebrates^2^. Another common method, still used today, is a double-staining procedure in which differential staining of cartilage with alcian blue is combined with bone staining with alizarin red S^3^. Various fixatives have been applied and tested for single or double-staining bone and/or cartilage, including ethanol, Bouin’s fixative, a mixture of acetic acid and ethanol, formalin, and paraformaldehyde^3–5^. In addition, several methods have been proposed for decolorization and clearing of the specimens, which are important steps in bone and cartilage staining. In Dawson’s procedure, specimens were decolored and cleared by treatment with 1% KOH^2^. Procedures using inorganic chemicals, such as KOH, are relatively simple and convenient because their potency for decolorization and cleaning soft tissues depends on their concentration, the reaction time, and temperature. It is therefore possible to adjust the degree of treatment and the schedule for the procedure as required. However, excessive treatment with a solution containing KOH can result in soft tissue becoming irreversibly dilapidated, which can make it difficult to retain the original orientation of each bone *in situ*. It is therefore important to carefully modify the condition of decolorization and cleaning with KOH according to the size and fixation of the sample. An alternative to cleaning with KOH is treatment with a protease such as trypsin^6^; however, these enzymes are more expensive and they require considerable attention to maintain optimal treatment conditions (such as temperature and pH). For fish, hydrogen peroxide (H_2_O_2_) can be used to bleach the soft tissue, although small air bubbles that result from the reaction with endogenous enzymes should be removed manually^7^. The Scale reagent, a urea-based agent used to make soft tissue transparent^8^, has also been used following bone staining with alizarin red S^9^; however, it can be difficult to use this solution for tests that require large-scale analysis, such as reproductive toxicology testing. To sum up, the optimal clearing procedure for bone staining remains to be established.

To prepare specimens with greater transparency, as much as possible of the skin, internal organs, muscles, and other soft tissues should be removed. However, this process frequently causes the severe destruction of skeletal structures. In addition, intermuscular bones are often lost while removing the skin and muscles of small fish. The destruction of skeletal structure, even the dislocation or loss of a small bone, could be disadvantageous, especially during developmental toxicity testing for chemicals or when screening for mutants. A bone staining procedure that clears the specimen without any artificial destruction would therefore provide a valuable tool, which would allow the establishment of a nondestructive bone staining protocol that retains small or incompletely calcified immature bones in their intact position at a microscopic level.

It takes a long time, typically several weeks or months, to complete cleared bone-stained specimens using the traditional protocol, even for small specimens such as small fish. Reducing the time taken would be of considerable benefit, especially for the testing of chemical toxicity, which requires the preparation and observation of a great many specimens. In the present study, we propose and demonstrate a new protocol for whole-mount bone staining in small fish (medaka and zebrafish) and *Xenopus* frogs. The procedure is rapid and relatively simple, and could be used to stain bones for a large number of specimens at once with no tissue destruction.

## Results

### Fixation and clearing

**Figure 1:**
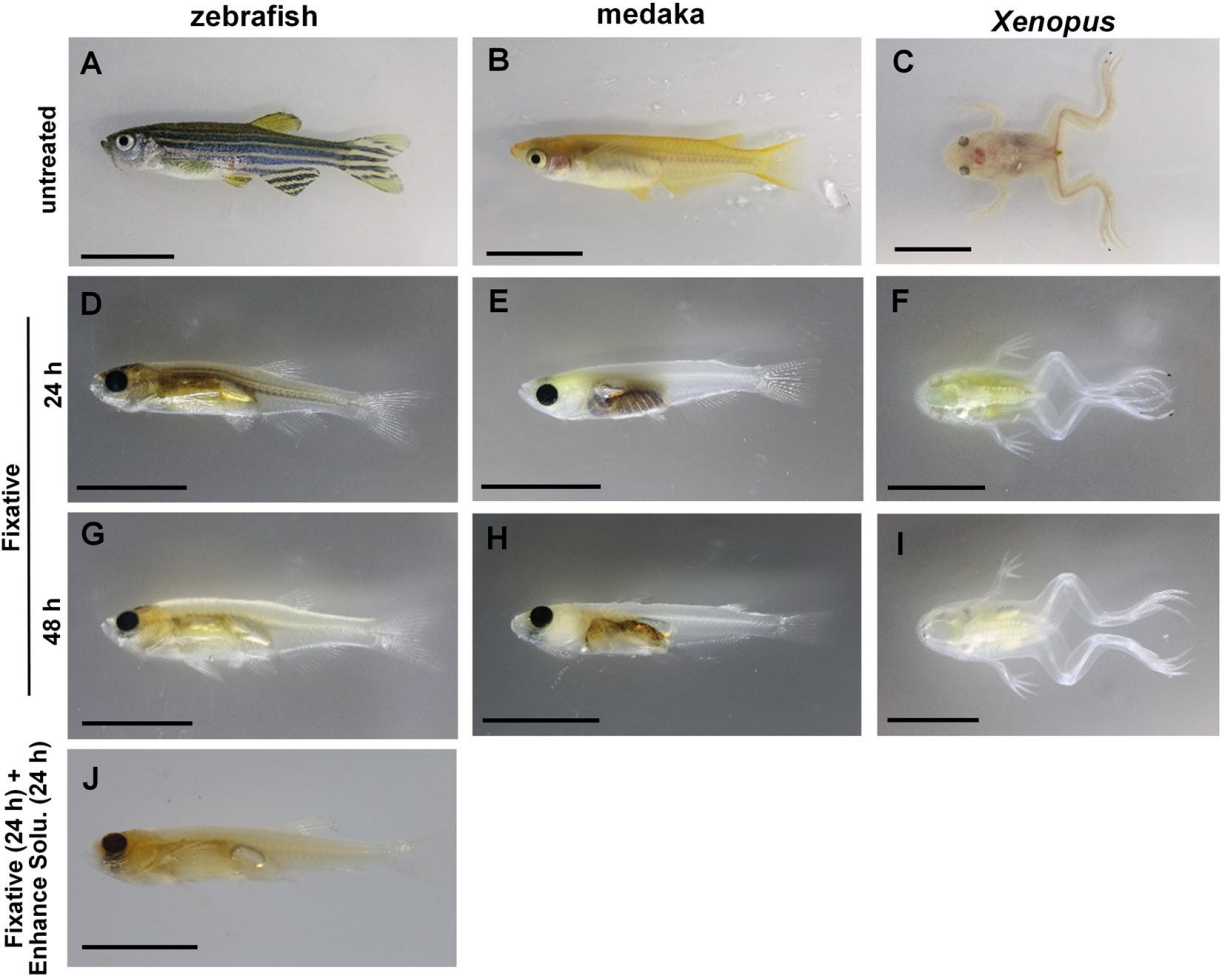
Efficacy of our new fixative. The medaka, zebrafish, and *Xenopus* after immersion in the new fixative for 24 h at 42 °C (**D–F**, respectively). These were decolored and cleared compared with untreated specimens (**A–C**, respectively). The decolorization and clearing increased with longer incubation in the fixative (**G–I**). After 48 h of incubation, the characteristic stripe pattern of the zebrafish disappeared (**H**). The zebrafish incubation in the enhancement solution for 24 h at 42 °C following immersion in the fixative for 24 h at 42 °C (**J**). Scale bar = 1 cm.

During incubation in our new fixative, the specimens bleached rapidly and became transparent (Fig. 1). Incubation for 24 h with the fixative at 42 °C rendered the medaka, zebrafish, and *Xenopus* translucent to enough to move to the next bone staining procedure (Fig. 1D–F, respectively). After incubation for 48 h, the medaka, zebrafish, and *Xenopus* became clearer (Fig. 1G–I, respectively), and it was possible to see the bones without staining. After immersion in the fixative for more than 3 days, the pigments in the peritoneum disappeared, but those in the eyes remained even when the specimens were incubated in the fixative for 1 week (see Supplementary Fig. S1). Longer incubation (for over 4 weeks or several months) caused the specimens to become completely transparent without the destruction of tissues (see Supplementary Fig. S1). In the *Xenopus*, the four limbs became transparent and the bones and joints could clearly be determined after immersion in the fixative for 24 h at 42 °C (Fig. 1F); longer incubation was needed to increase the transparency of the internal organs (Fig. 1I). The heme and/or its derivatives of blood also disappeared within 24 h in the fixative (see Supplementary Fig. S2).

To improve the efficiency of the decolorization and transparency process in the specimen that contained many pigments, such as zebrafish, we proposed an optional process, that is, incubation in the solution containing ethylene glycol and a nonionic surfactant with KOH. The specimens were incubated in this enhancement solution following which the black pigments, indicating the presence of melanin in melanophores, became lighter in color, especially on the body surface and in the eyes of the zebrafish (Fig. 1J and Supplementary Fig. S3). In the zebrafish, the pigments in the skull remained after treatment with only the fixative, but they disappeared after immersion into the enhancement solutions followed by treatment with the fixative (see Supplementary Fig. S3).

### Bone staining and observation with light microscopy

**Figure 2:**
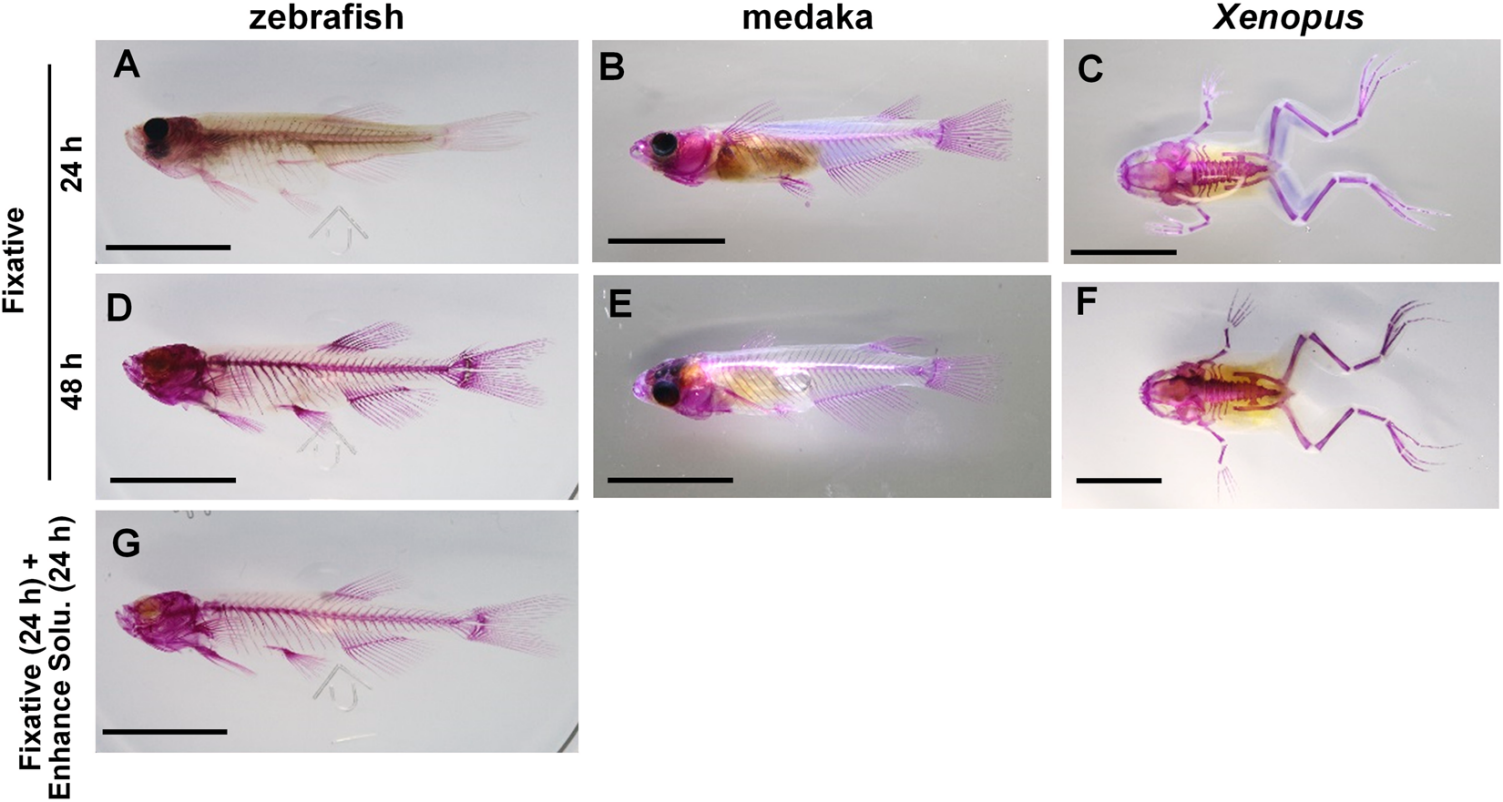
Whole-body images of the specimens after bone staining by the new procedure. The images show the medaka, zebrafish, and *Xenopus* incubated with the fixative for 24 h at 42 °C (upper row; **A–C**, respectively), incubated with the fixative for 48 h at 42 °C (middle row; **D**,**E**, and **F**, respectively), and incubated with fixative for 24 h at 42 °C followed by incubation with the enhancement solution for 24 h at 42 °C (lower row; **G**). Scale bar = 1 cm.

**Figure 3:**
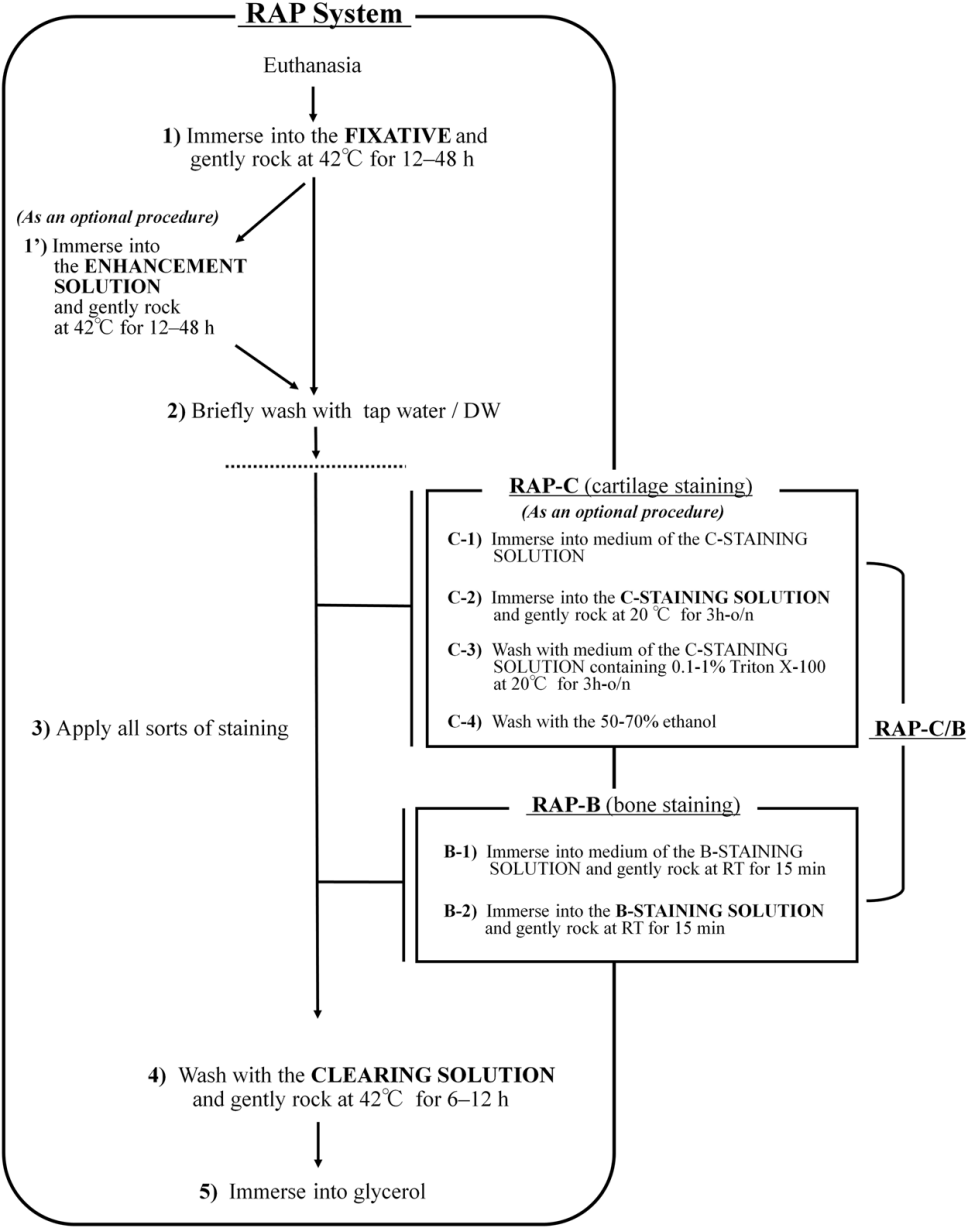
A flowchart of the rapid (RAP) system for bone staining (RAP-B). The new procedure for bone staining which is rapid, nondestructive, and allowed to obtain high-definition, fine bone-stained specimens, was based on our RAP system. Cartilage staining (RAP-C) can be applied as single staining or optionally added before the bone staining for double staining of bone and cartilage (RAP-B/C, Supplementary Fig. S8). **FIXATIVE:** 5% formalin, 5% Triton X-100, 1% potassium hydroxide (KOH); B-**STAINING SOLUTION:** 0.05% alizarin red S, 20% ethylene glycol, 1% KOH; **C-STAINING SOLUTION:** 50–70% ethanol, 20% acetate, 0.015–0.02% alcian blue; **CLEARING SOLUTION:** 20% Tween 20, 1% KOH; **ENHANCEMENT SOLUTION**: 20% ethylene glycol, 5% Triton X-100, 1% KOH.

**Figure 4:**
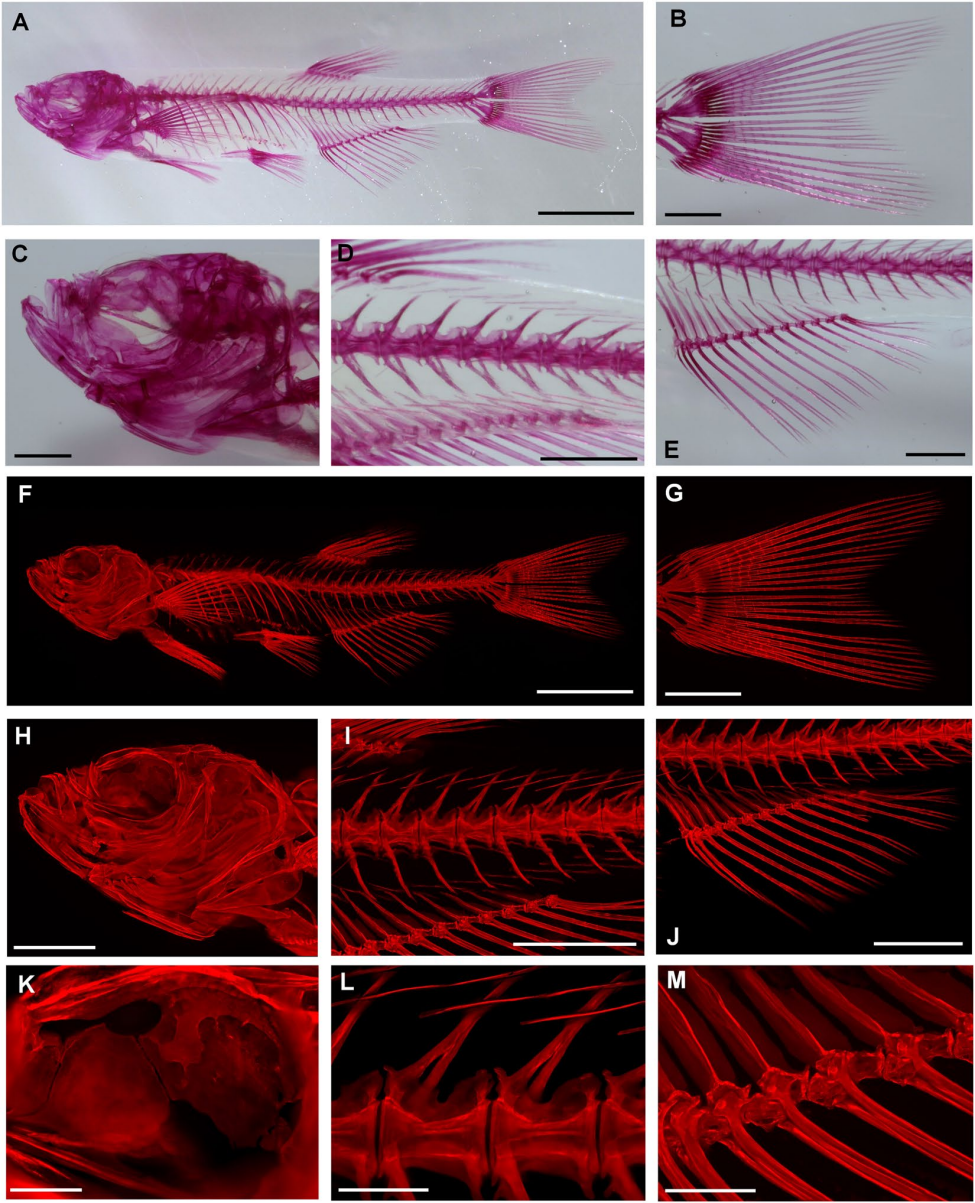
Whole-body (**A**) and magnification images of the tail fine ray (**B**), the skull (**C**), the vertebral column (**D**), and the anal fine ray (**E**) of the zebrafish after bone staining by our new procedure. Whole-body (**F**), and magnification fluorescence images the tail fine ray (**G**), the skull (**H**), the vertebral column (**I**), and the anal fine ray (**J**), and high- magnification fluorescence images of the orbit (**K**), the vertebral bones (**L**), and the joint part of proximal, intermedius, and distal pterygiophores of the anal fine ray (**M**) of the same zebrafish specimen shown in (**A–E**). The fluorescence images were reconstructed by maximum intensity projection (MIP) after image stitching. Scale bar in A and F = 5 mm; Scale bar in B, C, D, E, G, H, I, and J = 2 mm; Scale bars in K, L, and M = 0.5 mm.

After incubation with the fixative and enhancement solution, the specimens were stained with alizarin red S. Our staining solution containing alizarin red S and KOH with ethylene glycol accomplished specific staining in a short time. Even when the decolorization and transparency of the specimens was incomplete, for instance with the specimens incubated with only the fixative for 24 h (see Fig. 1D–F), the bones were stained well with alizarin red S (Fig. 2A–C, respectively). However, the staining was more clearly observed in the specimens with greater decolorization and transparency following longer incubation with the fixative (Fig. 2D–F) or incubation with the enhancement solution (Fig. 2G). A flowchart of the bone staining procedure is presented as Fig. 3. The entire skeletal system and magnified images of the zebrafish bone-stained with our new procedure are shown in Fig. 4.

### Observation with fluorescence microscopy

Also, fluorescence images were obtained for fluorescent of alizarin red S^10^. More clear images were provided by acquisition of the images with a fluorescence zoom microscope and reconstruction by maximum intensity projection after image stitching (Fig. 4F–M). In particular, high-magnification fluorescence images depicted detailed structures of each bone (Fig. 4K–M).

## Discussion

In this study, we proposed and demonstrated a new procedure of bone staining optimized for small fish and *Xenopus*. Our procedure is quite simple, rapid, and nondestructive, and should therefore be useful for examining the skeletogenesis and developmental toxicities of chemicals. One advantage of our protocol is the considerably shorter time to complete the procedure compared with previous methods (Supplementary Table S1). It required only 2 or 3 days to complete the bone staining for adult-sized medaka or zebrafish and an infant *Xenopus* (Supplementary Table S2). If a specimen had been transparentized very well before staining by keeping it in our fixative and/or by an extra immersion in the enhancement solution, the consequent staining and clearing process could be completed within a day. Another advantage of our procedure was that it resulted in less structural damage. Conventionl bone staining procedures require skinning and evisceration, with potential coincidental structural damage of the specimens, disruption of the original skeletal arrangement, and the loss of individual bones (Supplementary Fig. S4). On the other hand, our new protocol made the specimens completely clear without skinning or evisceration. This beneficial feature contributed to reducing the time and labor required for preparation of highly cleared bone stained specimens.

The most commonly used bone staining procedures comprise five steps: fixation, maceration with KOH, staining with alizarin red S, washing, and clearing with graded glycerol^11^. In our procedure, the specimens were first treated with the newly developed fixative, a mixture of formalin, Triton X-100, and KOH. This fixative allows the specimens to be fixed, decolorized, and transparentized at the same time. Combining these steps simplified and shortened the procedure. A noteworthy fact was that the morphology of the specimens was maintained even when the specimen was immersed in the fixative for a long time, although the fixative contained both KOH and Triton X-100 at high concentrations. This property of our fixative provides significant value because long-term immersion in a solution containing KOH and Triton X-100 at high concentrations usually lyses tissue and destroys the specimen; this has required careful attention to avoid missing specimens in the traditional bone staining procedure. This excellent property of the fixative contributes to expanding the application of the RAP system. To prepare the specimens of larger fishes or frogs for bone and/or cartilage staining, the samples are required to be immersed in the fixative until they became clear, as described below. In contrast to bone staining, which produces stable results between species, specimen-specific optimization is required to obtain the best results of double staining of bone and cartilage, depending on the size, stage of development, and species of specimens. It is also available for prenatal mammalian and avian fetuses and newborns soon after birth, although optimization of each procedure is needed. However, in newborns of avians and mammals after the stage at which feathers and hair begin to grow, specimens from whom the skin has not been removed are not sufficiently transparent with glycerol alone as the mounting solution. The next issue in this study is to develop new mounting reagents optimized for preparation of specimens of avians and mammals at postnatal stages.

Depending on the specimen’s size, the duration of incubation in our fixative would be needed to be changed in the range of only half day to several months (Supplementary Table S2), and longer preservation of the specimens in this fixative would be effective to reduce the time required for clearing after bone staining. However, if specimens are kept for more than several months, the pH of the fixative is reduced by formic acid generated by oxidation, potentially resulting in decalcification of bone and severe damage to the specimens. Therefore, during our bone staining procedure, the fixative must be changed several times until the specimens are decolored and well transparentized. It is also recommended that the fixative contains phosphate buffer (finally 50 mM) to avoid a decrease in pH of the fixative.

Triton X-100 may be effective for removing the brown/yellow color from the specimens, which is probably caused by heme and/or its derivatives, including bilirubin. Bilirubin is abundantly deposited in the tissue as complexes with other substances, such as albumin or collagen, and this interferes with clearing the tissues. Triton X-100 is able to remove heme and its derivatives under basic conditions, resulting in increased transparency of the specimens. Our fixative seems to be effective for eluting deposits of heme and its derivatives. It has previously been reported that CUBIC, a chemical cocktail containing amino alcohol for clearing the brain, elutes heme pigments from the tissue^12^.

Decolorization was incomplete in some parts, such as the eyes and skin of the zebrafish, even though the specimens were immersed in the fixative for more than 2 days. In this case, the enhancement solution was effective for removing the pigmentation. A possible mechanism for decolorization in our protocol is that KOH hydrolyzes the S-S bond of the melanin network, and then Triton X-100 removes it, similar to the process for decoloring hair. Ethylene glycol contained in the enhancement solution could increase the permeability of the cell membranes and consequently enhance the decolorization by KOH. However, excessive incubation with the enhancement solution could destroy the specimens.

Also, the bone staining solution for our protocol contained ethylene glycol, which would be effective for increasing the permeability of alizarin red S in this instance and consequently contribute to a reduction in the staining process. The shortened staining process could suppress nonspecific staining with alizarin red S in the soft tissues and thereby contribute to shortening of the subsequent washing process. Our clearing solution to remove excessive staining with alizarin red S was created with an ingenious combination of chemicals. The clearing solution contained Tween 20, which was a non-ionic surfactant similar to Triton X-100 but with higher cloud point. Tween 20 would help to percolate KOH into the specimens and thus reduce the washing time required. Replacing Tween 20 in the clearing solution with Triton X-100 caused the specimens to collapse.

Our highly transparentized bone staining specimens allowed clear florescence images of the skeletal system to be acquired. It was striking that with maximum intensity projection, it was possible to produce fine three-dimensional images of vertebral bones, intermuscular bones, and the skull including the orbits, surrounded by soft tissues (Fig. 4). The maximum intensity projection images clearly delineated the surface of each bone. Acquisition of fluorescence images by confocal microscopy allowed us to obtain high-definition fine images (Supplementary Fig. S5). It was even possible to clearly visualize bone that was surrounded by thick soft tissue in the highly transparentized specimens prepared using our rapid (RAP) system for bone staining (RAP-B) procedure (Supplementary Fig. S5). Thus, this system would facilitate the evaluation of detailed parts and detailed structures of each bone and be a powerful tool to detect abnormalities of skeletogenesis, even with only slight changes.

This bone staining procedure is possible to be developed to the multi-staining system for bone and other structures by combined it with immunostaining and/or genetic labeling procedures. However, most florescent proteins, such as green fluorescent protein, cannot be used with the present protocol because the highly basic KOH quenches the fluorescence activity. We are currently engaged in the development of a solution for clearing the tissue that allows the use of commonly used fluorochromes and fluorescent dyes. However, we have found that the nuclear staining with Hoechst 33342 was possible following bone staining by RAP-B procedure (Supplementary Fig. S6). Notably, details of histological structure were well retained in the specimens stained using the RAP-B procedure compared to specimens prepared using the conventional bone staining procedure (Supplementary Figs S6 and S7). This advantage of our nondestructive protocol suggests that the RAP system is applicable to a multi-colored staining procedure for bone and/or other structures, not only nuclei, in histological observations.

It was possible to apply our new bone staining procedure for double staining of bone and cartilage (Fig. 3 and Supplementary Fig. S8). However, staining cartilage with alcian blue would affect subsequent bone staining with alizarin red S. Thus, optimization of the staining condition was required before this experiment.

The new procedure that is rapid and nondestructive and allowed to obtain highly cleared specimens was based on our RAP system for bone staining of whole mount specimens (Fig. 3). In the beginning of a series of procedures, the specimens were fixed, decolorized, and transparentized using a newly developed fixative containing formalin and Triton-X 100 with KOH. Subsequently, it was possible to apply other staining procedure, such as RAP-B, cartilage staining (RAP-C), or double staining with cartilage and bone (RAP-B/C). In the present study, we proposed a RAP system optimized for small fish and *Xenopus*. However, whole-mount bone staining using our RAP system is also possible to apply to larger specimens, such as rodents, and to a batch staining system for a large-scale analysis. RAP system that is quite simple, rapid, and nondestructive, therefore, would be expected to be widely used as a standard procedure for bone staining in testing the developmental toxicity of chemicals and for screening for knockout or mutant animals.

## Materials and Methods

### Study approval

All procedures in this study were approved by Institutional Animal Care and Use committee of Kanazawa Medical University (No. 2017-7) and performed in accordance with the guidelines for animal experiments based on Japanese guidelines and provided by the Ethics Committee of Kanazawa Medical University.

### Fishes and amphibians

Adult Japanese medaka (*Oryzias latipes*, 3–4-cm body length) and zebrafish (*Danio rerio*, 3–4-cm body length) were obtained from Meito Suien (Nagoya, Japan). The fish were reared at 25 °C ± 2 °C with a 14-h/10-h light/dark cycle. *Xenopus laevis* frogs (3–4-cm body length) were obtained from Watanabe Zousyoku (Yachiyo, Hyogo, Japan) and housed until use at 18 °C ± 2 °C with a 12-h/12-h light/dark cycle.

### Fixation and clearing

The fish and amphibians were kept on ice for 10–60 min as anesthesia and then immersed into a freshly prepared fixative consisting of a mixture of 5% formalin (formalin neutral buffered solution, Wako, Osaka, Japan), 5% polyoxyethylene (10) octylphenyl ether (equivalent to Triton X-100, Wako, Osaka, Japan), and 1% KOH (Wako, Osaka, Japan) and rocked for 12 h or longer at 42 °C or room temperature (RT). Following incubation in the fixative, some specimens were immersed for clearing into an enhancement solution containing 20% ethylene glycol (Wako, Osaka, Japan), 5% polyoxyethylene (10) octylphenyl ether, and 1% KOH. The scales of the medaka and zebrafish were removed using cotton buds under a binocular microscope.

### Bone staining

The specimens were washed briefly with a solution containing 20% ethylene glycol and 1% KOH, then incubated in a freshly prepared bone staining solution containing 0.05% alizarin red S (Wako, Osaka, Japan), 20% ethylene glycol, and 1% KOH at RT for 30 min with gentle agitation. Over-stained specimens with alizarin red S were thoroughly washed in a prewarmed clearing solution containing 20% polyoxyethylene (20) sorbitan monolaurate (the equivalent of Tween 20, Wako, Osaka, Japan) and 1% KOH at 42 °C for 3 h or more with agitation. After the background coloration was removed, the specimens were moved through a graded series of glycerol (50% to 90%) and then kept in 100% glycerol.

### Observation

The specimens were observed with a binocular microscope (Stemi DV4, Zeiss, Oberkochen, Germany) and photographed with a EOS 60D digital camera (Canon, Tokyo) equipped with a macro lens (EF-S60 mm F2.8, Canon, Tokyo). For fluorescent observation, an Axio Zoom.V16 microscope equipped with an AxioCam HRm camera (Zeiss, Oberkochen, Germany) was used. Panorama images of the specimens were produced using Zen 2013 imaging software (Zeiss, Oberkochen, Germany).

### Data availability

All data generated or analyzed during this study are available from the corresponding author on reasonable request.

## Electronic supplementary material

Supplementary Information
