# Author Correction: A rapid and nondestructive protocol for whole-mount bone staining of small fish and *Xenopus*

**DOI:** 10.1038/s41598-018-36375-3

**Published:** 2018-12-12

**Authors:** Hiromi Sakata-Haga, Maimi Uchishiba, Hiroki Shimada, Tsuyoshi Tsukada, Mayumi Mitani, Tomohiro Arikawa, Hiroki Shoji, Toshihisa Hatta

**Affiliations:** 1 Department of Anatomy, Kanazawa Medical University, Ishikawa, Japan; 2 Department of Obstetrics and Gynecology, Wakayama Medical University, Wakayama, Japan; 3 Department of Biology, Kanazawa Medical University, Ishikawa, Japan

Correction to: *Scientific Reports* 10.1038/s41598-018-25836-4, published online 10 May 2018

In this Article, the legend of Figure 3 is incorrect:

“A flowchart of the rapid (RAP) system for bone staining (RAP-B). The new procedure for bone staining which is rapid, nondestructive, and allowed to obtain high-definition, fine bone-stained specimens, was based on our RAP system. Cartilage staining (RAP-C) can be applied as single staining or optionally added before the bone staining for double staining of bone and cartilage (RAP-B/C, Supplementary Fig. S8). FIXATIVE: 5% formalin, 5% Triton X-100, 1% potassium hydroxide (KOH); B-STAINING SOLUTION: 0.05% alizarin red S, 20% ethylene glycol, 1% KOH; C-STAINING SOLUTION: 50–70% ethanol, 20% acetate, 0.015–0.02% alcian blue; CLEARING SOLUTION: 20% Tween 20, 1% KOH; ENHANCEMENT SOLUTION: 20% ethylene glycol, 5% Triton X-100, 1% KOH.”

should read:

“A flowchart of the rapid (RAP) system for bone staining (RAP-B). The new procedure for bone staining which is rapid, nondestructive, and allowed to obtain high-definition, fine bone-stained specimens, was based on our RAP system. Cartilage staining (RAP-C) can be applied as single staining or optionally added before the bone staining for double staining of bone and cartilage (RAP-B/C, Supplementary Fig. S8). FIXATIVE: 5% formalin, 5% Triton X-100, 1% potassium hydroxide (KOH); B-STAINING SOLUTION: 0.05% alizarin red S, 20% ethylene glycol, 1% KOH; C-STAINING SOLUTION: 50–70% ethanol, 20% acetic acid, 0.015–0.02% alcian blue; CLEARING SOLUTION: 20% Tween 20, 1% KOH; ENHANCEMENT SOLUTION: 20% ethylene glycol, 5% Triton X-100, 1% KOH.”

