# Supplementary material for: A rapid and nondestructive protocol for whole-mount bone staining of small fish and Xenopus
Source: Sci Rep. 2018 May 10;8:7453. doi: 10.1038/s41598-018-25836-4 (PMC5945591; doi:10.1038/s41598-018-25836-4)
Supplement: Supplementary file 1 — Supplementary Information [file 41598_2018_25836_MOESM1_ESM.pdf]

Supplementary Information

A rapid and nondestructive protocol for whole-mount bone staining of small fish and *Xenopus*

Hiromi Sakata-Haga[1], Maimi Uchishiba[2], Hiroki Shimada[1], Tsuyoshi Tsukada[1], Mayumi Mitani[1], Tomohiro Arikawa[3], Hiroki Shoji[3], & Toshihisa Hatta[1*]

[1] Department of Anatomy, Kanazawa Medical University, Ishikawa, Japan

[2]Department of Obstetrics and Gynecology, Wakayama Medical University, Wakayama Japan

[3]Department of Biology, Kanazawa Medical University, Ishikawa, Japan

[*]Correspondence and requests for materials should be addressed to T.H (e-mail: thatta@kanazawa-med.ac.jp)

Supplementary Figures

Figure S1

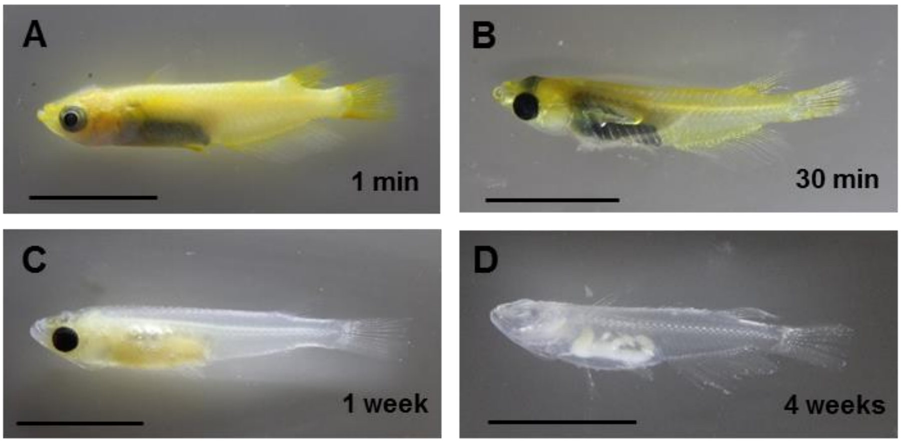

Figure S1. Change in the color and transparency over time of a medaka immersed in our new fixative. The medaka was immersed in the fixative at 42 °C for 1 or 4 weeks at 42 °C (A, B, C and D, respectively). Scale bar = 1 cm.

Figure S2

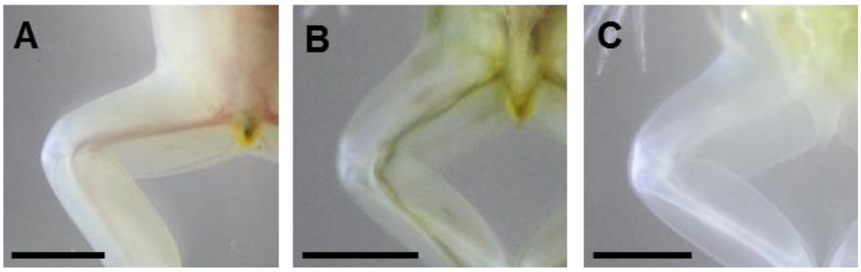

Figure S2. Fixative efficacy for removal of heme and its derivatives. Blood in the vessels was clearly visible in the thigh of an untreated *Xenopus* (A). Following incubation in our new fixative at 42 °C for 30 min, blood in the vessels changed color (B). After incubation at 42 °C for 24 h, the blood lost its color and was no longer invisible (C). Scale bar = 0.5 cm.

Figure S3

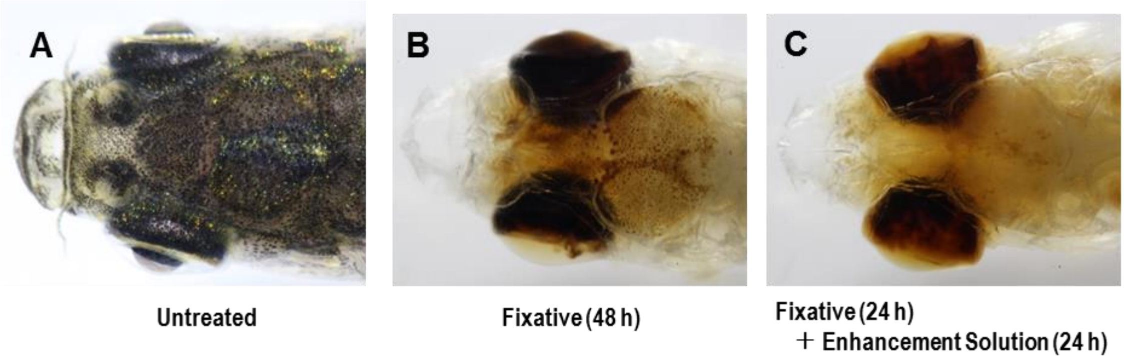

Figure S3. Efficacy of the enhancement solution in removing black pigment from the skull. Abundant black pigment was observed in the skull of an untreated zebrafish (A). Following incubation in our new fixative for 48 h at 42 °C, the black pigment remained (B). Following incubation with the fixative for 24 h at 42 °C, followed by the enhancement solution for 24 h at 42 °C, the black pigment was no longer visible (C).

Table S1

Table S1. Schedule comparison of the RAP-B system and a conventional procedure* for bone staining in fish. Photographs of medaka in each step are shown.

	RAP system	Conventional procedure
Day 1	Fixation/bleaching	Fixation
	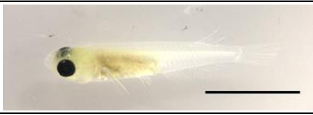	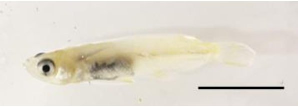
	Rinse	Rinse
Day2	Staining	Evisceration and skining
	Washing	Bleaching
	Clearing with glycerol	
	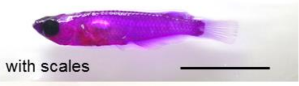 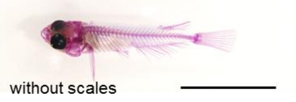	
Day3		Rinse
		Bleaching
		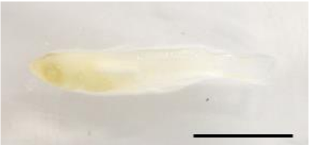
Day4		Staining
Day5		Rinse
		Clearing with trypsin
Day6		Clearing with glycerol
		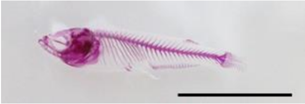

* Westerfield M. The zebrafish book, 5th edition; A guide for the laboratory use of zebrafish (*Danio rerio*). Eugene, University of Oregon Press (2007).

Table S2. Modification examples of the RAP-B procedure. Immersion duration in the fixative and enhancement solution depends on the fish size and species of fish. Solution replacement every 12–24 h is recommended.

			Step1-1	Step 1-2 (Optional)	Step 3	Step 4	Step 5	Total
Length (mm)	Fish species	Evisceration and skinning	Fixation	Enhancement	Staining	Washing	Clearing	
15-20	Zebrafish	No	> 12 h	> 12 h	0.5-1 h	6 h	> 6 h	< 2 days
20-25	Medaka	No	> 12 h	unnecessary	0.5-1 h	6 h	> 6 h	< 1 day
> 26	Medaka	No	> 24 h	unnecessary	0.5-1 h	6 h	> 6 h	< 2 days
> 35	Zebrafish	Yes	> 12 h	> 12 h	0.5-1 h	6 h	> 6 h	< 2 days
> 35	Zebrafish	No	> 24 h	> 24 h	0.5-1 h	6 h	> 6 h	< 3 days
> 70	Goldfish	Yes	> 2 days	> 1 day	0.5-1 h	6 h	> 12 h	< 4 days
> 70	Goldfish	No	> 4 days	> 4 days	0.5-1 h	6 h	> 12 h	< 10 days

Figure S4

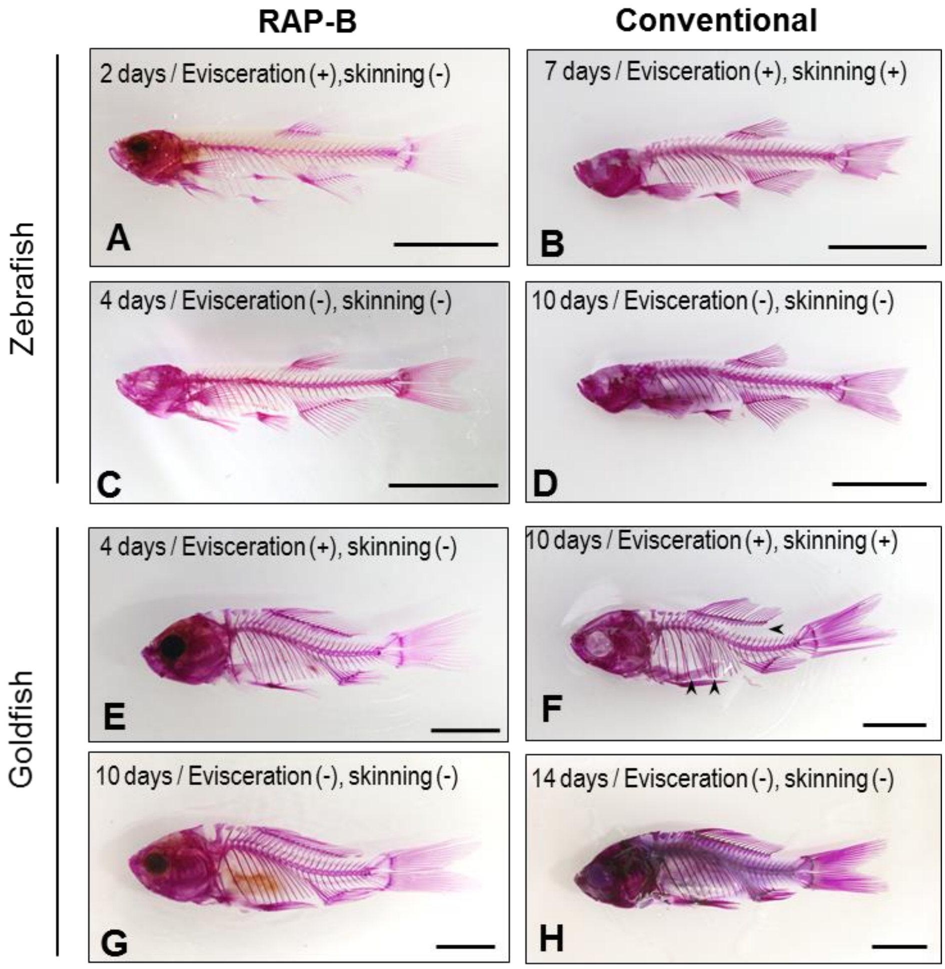

Figure S4. Comparison of the schedule between the RAP-B system and the conventional procedure for bone staining in fish. Despite the shorter preparation time, specimens prepared using the RAP-B procedure were clearer than those prepared using the conventional bone staining procedure. Specimens prepared using the conventional procedure were difficult to be transparented without skinning and evisceration (D and H), even if it ha longer days compared to the specimens using the RAP-B system (C and G). It might be possible to be transparented by longer treatment, however it would induce loss of stiffness. Furthermore, owing to the treatments of evisceration, skinning and trypsin digestion, disorder of the placement of skeleton has often occurred with the conventional protocol (arrow heads in F). Maintained stiffness is also an important merit of the specimens by RAP-B procedure. Scale bar = 1 cm

Figure S5

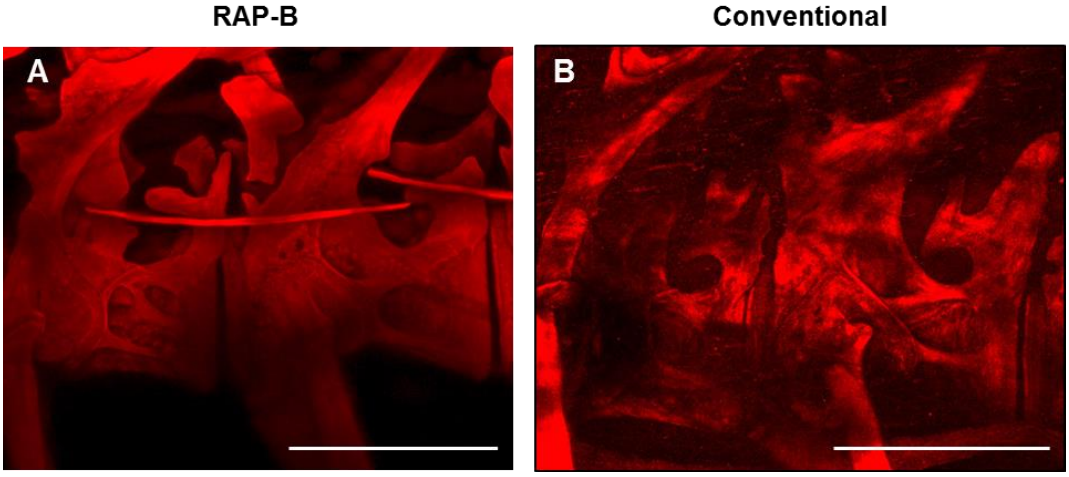

Figure S5. Fluorescent MIP images of abdominal vertebrae in zebrafish bone-stained using RAP-B or the conventional procedure. The MIP images were generated by the image processing software Zen (Zeiss) based on optical slice images acquired every 10 μm across 1,500 μm thickness using a confocal laser scanning microscopy (LSM 710, Zeiss). MIP images of the RAP-B specimen (A) exhibited a more precise bone shape, even in the abdominal vertebrae surrounded by thick soft tissue, compared to that of the conventional procedure (B). Scale bar = 500 μm.

Figure S6

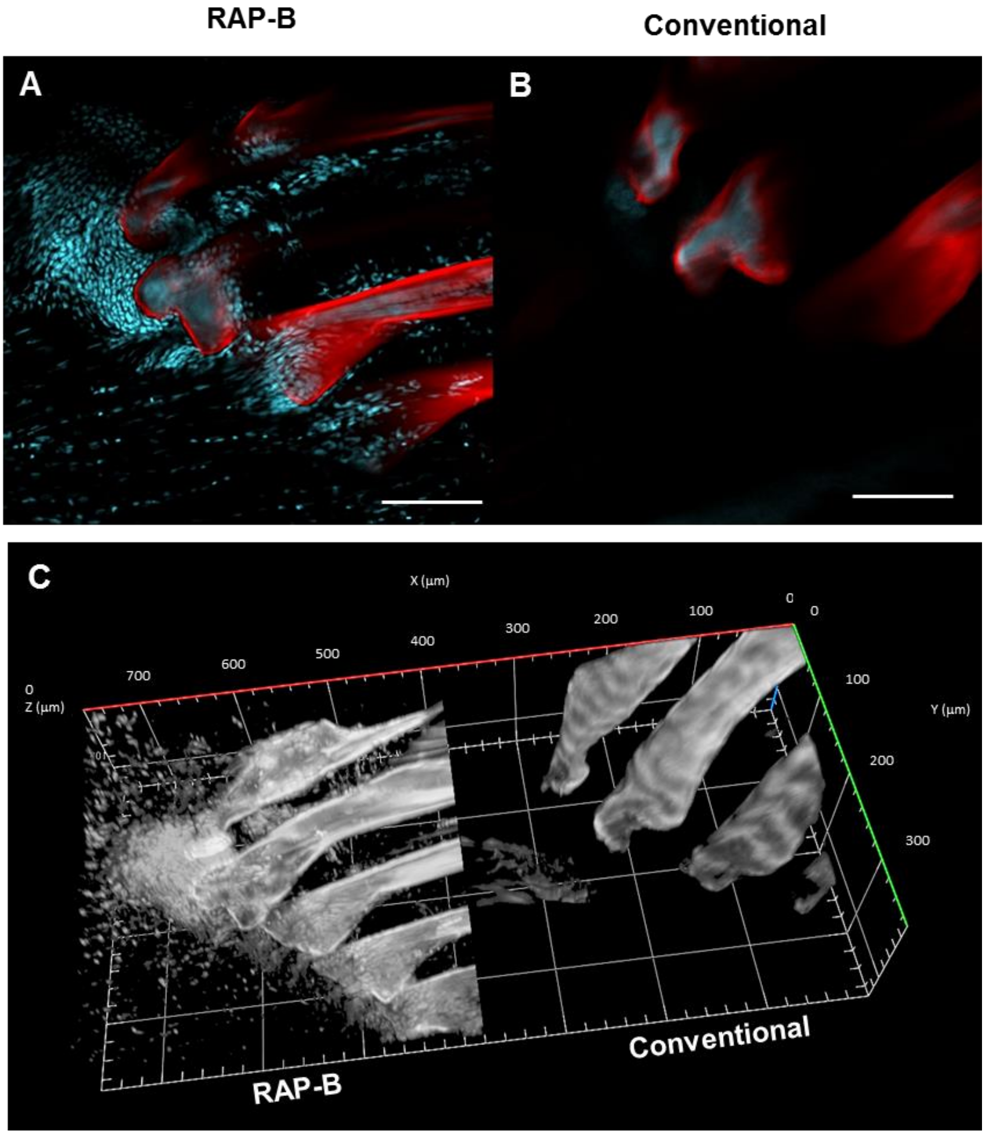

Figure S6. Fluorescent optical slice images (A and B) and 3D images (C) at the root of the pectoral fin in medaka. Whole-mount medaka specimens were stained with Hoechst 33342 following bone staining by RAP-B or the conventional procedure. The 3D image was generated using the image processing software ZEN (Zeiss) based on optical slice images acquired every 10 µm across 140 µm thickness using a confocal laser scanning microscopy (LSM 710, Zeiss). Nuclei were clearly detected in the RAP-B specimen (A), but only a small number of indistinct nuclei were detected in the specimen bone-stained using the conventional procedure (B). In the 3D image, it was more clearly confirmed that nuclei were remained in the tissue of the RAP B specimen, but not in the tissue of the specimen stained using conventional procedure. Red: alizarin, Blue; Hoechst 33342 in A and B. Scalebar = 50 µm

Figure S7

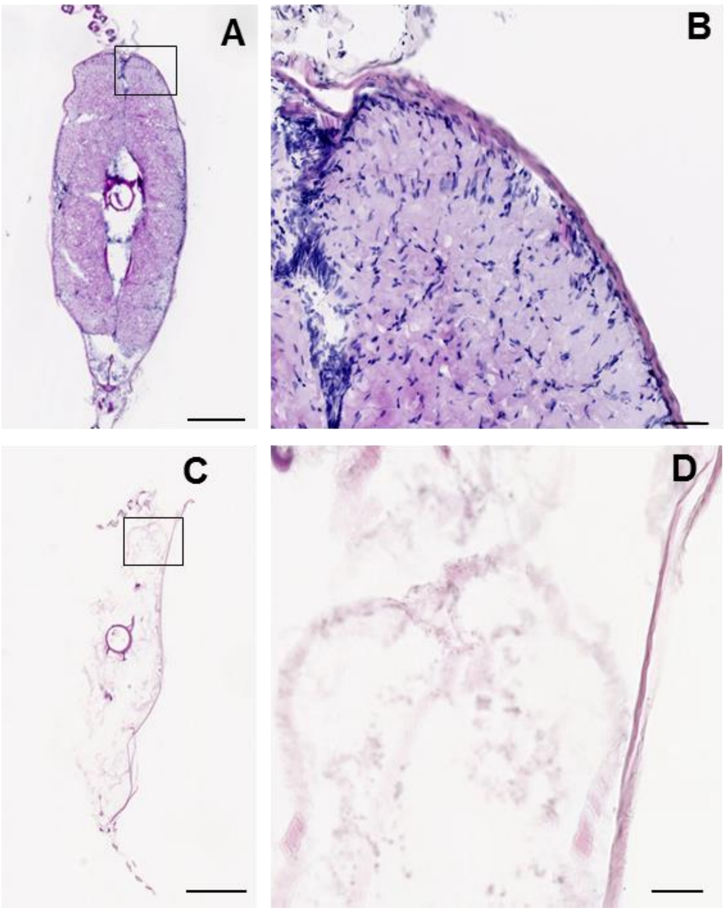

Figure S7. Axial frozen sections (20 μm thickness) of zebrafish were prepared following whole mount bone staining by RAP-B (A and B) or the conventional procedure (C and D). The sections were stained with hematoxylin and eosin. Photographs were obtained using a digital slide scanner (NanoZoomer C9600-03, Hamamatsu Photonics). Histological structure in the RAP-B specimens appeared be well retained (B). In contrast, specimens treated by the conventional protocol showed severe destruction of tissues (D). Scale bar in A and C = 500 μm, Scale bar in B and D = 50 μm

Figure S8

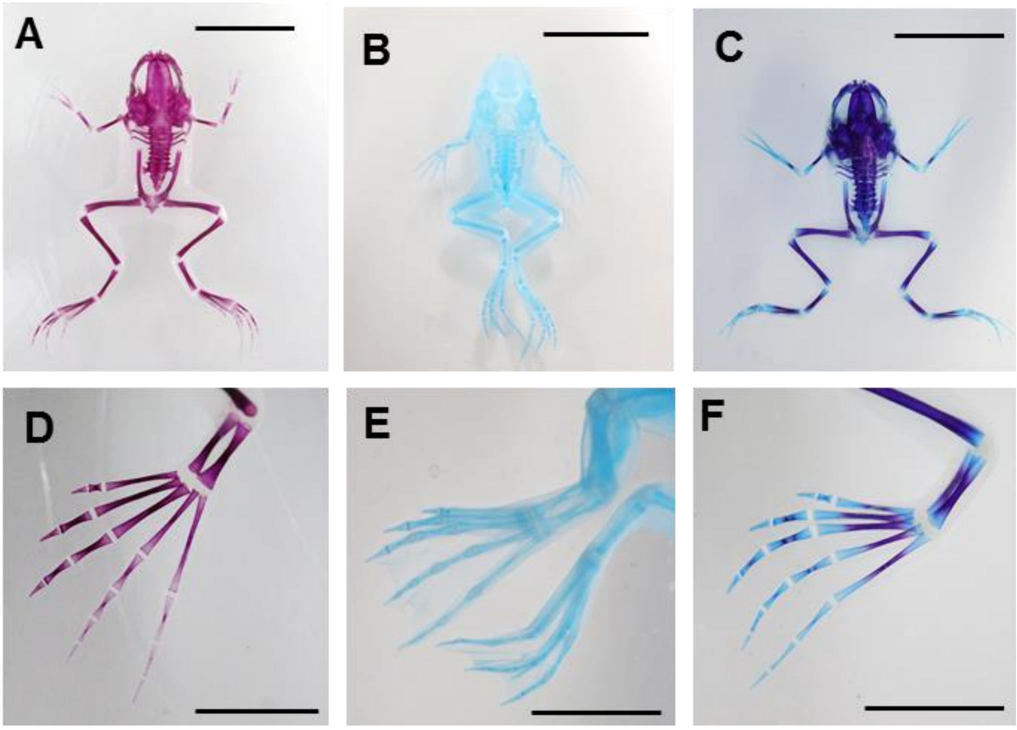

Figure S8. Application of the RAP system to cartilage staining. Xenopus transparentized using the RAP system were single stained with alizarin red S for bone (RAP-B; A and D) or alcian blue 8GX for cartilage (RAP-C; B and E), and double stained with alcian blue and alizarin (RAP-C/B; C and F). Scale bar in A, B, and C = 1 cm, Scalebar in D, E, and F = 0.5 cm
